# Headache

**Published:** 1896-05

**Authors:** C. M. Boger

**Affiliations:** Parkersburg, W. Va.


### HEADACHE.

C. M. BOGER, M. D., PARKERSBURG, W. VA.

Mrs. Margaret C., æt. thirty, came under my care September 6th, 1894, with the following history: Inclined to obesity; bilious, lymphatic temperament; headache first appeared at puberty; it either precedes or follows the menses; as a child had stomach disorders and was generally weak; pains are not well defined as to character, but change place over head; it is accompanied by vomiting of bile, and followed by jaundice and stiffness of the neck, always lasting several weeks; great intolerance of heat and pressure of the neck-band; bad taste in morning. ℞ Lach.$^{cm}$ and Sac-lac.

October 24th, much better. December 31st, slight return of symptoms. ℞ Lach.$^{cm}$ and Sac-lac. October 11th, 1895, reports that she is well, and has had no headache since the last prescription. The chief interest in this case centres in the fact that two doses of a high potency cured a headache which had persisted fifteen years, and was evidently purely of a reflex nature.

